# Extracellular Matrix Rigidity-dependent Sphingosine-1-phosphate Secretion Regulates Metastatic Cancer Cell Invasion and Adhesion

**DOI:** 10.1038/srep21564

**Published:** 2016-02-15

**Authors:** Panseon Ko, Daehwan Kim, Eunae You, Jangho Jung, Somi Oh, Jaehyun Kim, Kwang-Ho Lee, Sangmyung Rhee

**Affiliations:** 1 Department of Life Science, Chung-Ang University, Seoul, 06974, Republic of Korea

## Abstract

Dynamic interaction between cancer cells and the surrounding microenvironment is critical for cancer progression via changes in cellular behavior including alteration of secreted molecules. However, the molecular mechanisms underlying the influence exerted by the cancer microenvironment on secretion of molecules during cancer progression remain largely unknown. In this study, we report that secretion of spingsine-1-phosphate (S1P) and its regulator, *SphK1* expression is dependent of the substrate rigidity, which is critical for the balance between cancer cell invasion and adhesion. Conditioned media (CM) of MDA-MB-231, an aggressive breast cancer cell obtained from soft substrate (~0.5 kPa) induced chemo-attractive invasion, while CM obtained from stiff substrate (~2.5 kPa) increased cell adhesion instead. We found that the expression of *SphK1* is upregulated in the stiff substrate, resulting in an increase in S1P levels in the CM. We also found that upregulation of *SphK1* expression in the stiff substrate is dominant in metastatic cancer cells but not in primary cancer cells. These results suggest that alterations in the mechanical environment of the ECM surrounding the tumor cells actively regulate cellular properties such as secretion, which in turn, may contribute to cancer progression.

Cancer metastasis is a complicated process by which tumor cells spread from the primary site and invade the surrounding extracellular matrix (ECM). The invading cells enter the bloodstream, which enables them to spread quickly and efficiently to distant sites within the body, where they extravasate from the vasculature to colonize the metastatic sites^1^,^2^. The altered secretory pattern of cancer cells is the key mediator for promoting invasion and metastasis^3^,^4^. For example, several secreted cytokines including transforming growth factor-β (TGF-β) and metalloproteinases are well characterized as factors that enhance cancer cell growth, stromal interaction, and metastasis in breast cancer^5^,^6^,^7^. Moreover, these secreted factors are not only involved in cancer cell invasion but also regulate the colonization of cancer cells at the secondary site^8^.

It has been reported that dynamic changes in the stromal microenvironment within breast cancer tissues is critical for cancer progression^9^,^10^. Specifically, biophysical properties of the stroma surrounding breast cancer cells are key indicators of breast cancer progression. During tumorigenesis, normal stroma transforms into activated stroma, which is typically stiffer; breast cancer tissue can be ten times more rigid than normal breast tissue^11^,^12^. Increased ECM stiffness enhances and promotes cell growth, survival, and migration^13^. Moreover, ECM rigidity influences disruption of tissue morphogenesis by increasing cell tension, gene expression and secretion^14^. On stiff substrates, ECM molecules such as collagen IV, fibronectin, and perlecan are downregulated and secreted to a lesser extent in endothelial cells^15^. However, the complex biological relationship between the microenvironment-mediated autocrine materials and alteration of the environment by active factors secreted by cells during cancer progression remains poorly understood.

Accumulating evidence indicates that bioactive lipids such as lysophosphatidic acid (LPA) and sphingosine-1-phosphate (S1P) contribute to malignant progression in lung, colon, prostate, and breast carcinogenesis in a paracrine and/or autocrine manner^16^,^17^. S1P generated by sphingosine kinase 1 (SphK1) is secreted by the cell via ABCC1 transport and binds to the S1P receptor (S1PR) to promote cellular proliferation, migration, and contraction^18^,^19^,^20^. NIH3T3 fibroblasts overexpressing SphK1 acquired the transformed phenotype, including colony growth in soft agar and the ability to form tumors in NOD/SCID mice^21^. In addition, level of SphK1 is upregulated in various forms of cancer including breast cancer^18^,^22^ and correlates with poor prognosis^23^ and resistance to chemotherapy^24^. Several heterotrimeric, G-protein-coupled receptors have been identified as S1PRs, and their presence determines the differential cellular function of S1P^25^,^26^. However, for the aggressive breast cancer cell line MDA-MB-231, S1P shows anti-migratory and invasive effects in a receptor-independent manner, via an unknown molecular mechanism^27^.

In this study, we compared the effect of conditioned medium (CM) derived from MDA-MB-231 human breast cancer cells (MDA-CM) and MCF10A normal breast epithelial cells (10A-CM) on cell migration and invasion using the collagen-coated Transwell system. The results indicated that the serum-induced migration and invasion of MDA-MB-231 cells was significantly decreased by MDA-CM. CM produced in the presence of pharmacological inhibitors of protein secretion and exosome formation did not rescue the inhibitory function of MDA-CM. However, depleting the lipid growth factor from MDA-CM by activated charcoal as well as CM obtained from cells with siRNA-mediated *SphK1* silencing did not show inhibition of cell invasion. We also found that *SphK1* expression is upregulated in breast tumors with increased stiffness (approximately 2.5 kPa) compared with that in normal breast tissue (approximately 0.5 kPa). Additionally, MDA-MB-231 cell invasion was unaffected by CM obtained from cells cultured on soft matrix, whereas CM obtained from stiff matrix seemed to promote cell adhesion. Finally, regulation of *SphK1* expression and S1P secretion by ECM stiffness is dependent on cancer cell origin. In primary cell lines, increasing ECM stiffness reduced *SphK1* expression. On the contrary, in aggressive metastatic cell lines, increasing ECM stiffness induced *SphK1* expression. Additionally, CM harvested from cells with upregulated *SphK1* expression cultured on stiff or soft matrix enhanced cell adhesion. Thus, our data suggest that the temporal regulation of S1P secretion by the differential mechanical conditions is one of key regulators of the balance between cancer cell invasion and adhesion during metastasis.

## Results

### CM derived from MDA-MB-231 cells shows an inhibitory effect on invasion and migration

**Figure 1:**
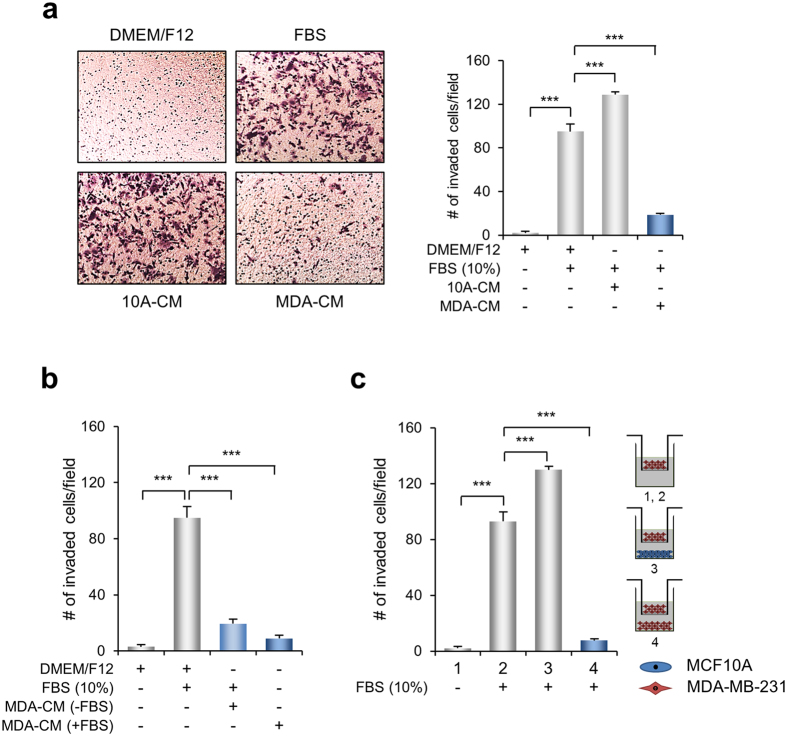
MDA-CM inhibits serum-induced chemo-attractive invasion. Invasion of MDA-MB-231 cells was examined with the MDA- or 10A-CM using the Transwell invasion assay system for 24 h. (**a**) MDA- and 10A-CM were prepared by incubating for 24 h in serum-free media. Left panels, representative images of invading cells. Right panels, quantification of the average number of invading cells per photographic field shown in the left panel. Values represent mean ± standard error of the mean (s.e.m.), ***p < 0.001. (**b**) As the right panel in Fig. 1a, except that Transwell invasion was performed with MDA-CM, prepared from MDA-MB-231 cell culture with or without serum. Values represent mean ± s.e.m., ***p < 0.001. (**c**) MDA-MB-231 and MCF10A cells (1 × 10^5^ cells/well) were placed at the bottom of the lower chamber, as shown in the right panels in a schematic diagram of the co-culture chemotaxis assay. The average number of invading cells/photographic field was counted. Values represent mean ± s.e.m., ***p < 0.001.

To test the effect of molecules secreted from cancer cells on cell invasion and migration, we compared the ability of CM derived from MDA-MB-231, an aggressive breast cancer cell line and MDF10A, a normal breast epithelial cell line, on invasion of MDA-MD-231 cells. A cell migration and invasion assay using the non-collagen-coated or collagen-coated Transwell system showed that chemo-attractive invasion of MDA-MB-231 cells was significantly increased when serum was loaded in the lower chamber. 10A-CM appeared to increase cell migration and invasion slightly compared to the control conditions. However, MDA-CM decreased cell migration and invasion significantly (Fig. 1a and Supplementary Fig. S1).

MDA-CM was obtained from the cells incubated for 24 h in a serum-free medium in which cells are likely to induce self-decomposition. To rule out this possibility, MDA-CM was prepared with serum and its ability to induce cell invasion was examined. Figure 1b shows that chemo-attractive invasion of MDA-MB-231 cells was significantly inhibited in the MDA-CM prepared with serum. Thus, these results indicate that the molecules secreted from MDA-MB-231 cells negatively regulate cell migration and invasion.

To further confirm whether the factors secreted from MDA-MB-231 cells directly inhibited cell invasion, the Transwell invasion assay was carried out, where MCF10A or MDA-MB-231 cells were seeded in the lower chamber. Interestingly, MCF10A cells seeded in the lower chamber showed a similar extent of cell invasion to 10A-CM. However, the invasion of MDA-MB-231 cells while MDA-MB-231 cells were present in the lower chamber was dramatically inhibited (Fig. 1c). These results clearly demonstrate that the molecules secreted from MDA-MB-231 cells contain inhibitory factor/s for cell invasion.

### MDA-MB-231 cell-derived CM induced focal adhesion and cellular contractility

**Figure 2:**
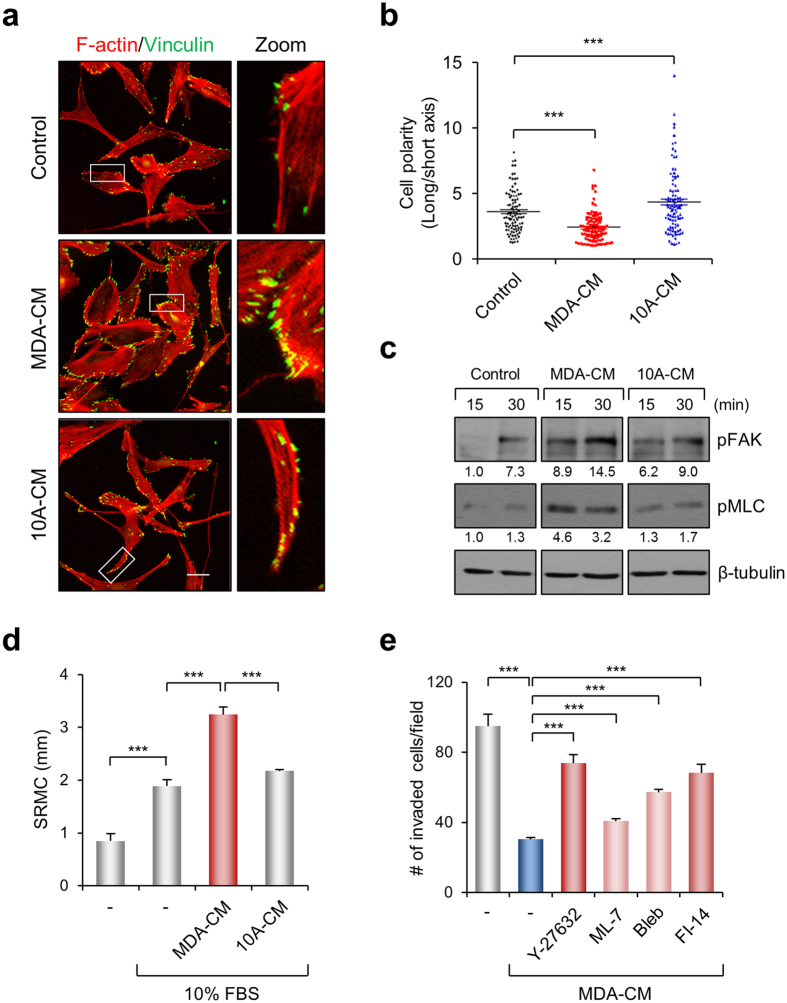
MDA-CM promotes focal adhesion formation and cell contractility. (**a**) MDA-MB-231 cells were cultured on collagen-coated coverslips for 24 h with MDA- or 10A-CM. After incubation, cells were stained for actin and focal adhesions. Scale bar, 30 μm. (**b**) Cells shown in (**a**) were analyzed for cell polarity by measuring the ratio of the long axis divided by the short axis. For each value, measurements were made for approximately 100 cells. ***p < 0.001. (**c**) MDA-MB-231 cells were incubated with MDA or 10A-CM for the indicated times. Cell lysates were immunoblotted with anti-pFAK, pMLC, and α-tubulin antibodies. (**d**) SRMC was carried out for 1 h in MDA- or 10A-CM containing serum as indicated. Matrix contraction values shown are the mean ± s.e.m. of triplicate samples. ***p < 0.001. (**e**) MDA-MB-231 cells were treated with inhibitors of Rho kinase (Y-27632, 10 μM), MLCK (ML-7, 10 μM), Myosin IIA (Blebbistatin, 10 μM), and FAK (FI-14, 10 μM), and Transwell invasion assays were performed with MDA-CM. Values represent mean ± s.e.m., ***p < 0.001.

It has been previously reported that invading cells possess a slender morphological form with numerous lamellipodia and weak stress fiber formations^28^. We attempted a comparison of morphological characteristics of MDA-MB-231 cells in response to MDA- or 10A-CM treatment. Figure 2a,b show representative images of cells and statistical analysis of cell polarity with MDA- and 10A-CM, respectively. Most of the cells treated with MDA-CM possessed a round form with developed actin stress fibers, whereas 10A-CM induced the cells to acquire more slender and elongated morphologies. In addition, MDA-CM-induced vinculin-positive focal adhesions along the cell periphery, while cells under 10A-CM did not develop focal adhesions or stress fibers (Fig. 2a). Furthermore, immunoblotting for pFAK and pMLC showed that the cells treated with MDA-CM profoundly induced the phosphorylation of FAK and MLC compared to control cells or 10A-CM (Fig. 2c). Thus, these results suggest that MDA-CM induces cellular contractility resulting from the development of focal adhesion and stress fibers.

The increase in cell contractility with MDA-CM was confirmed by the measurement of stress-related collagen matrix contraction (SRMC) which is a typical method for evaluating cell contractility induced by mechanical tension^29^. SRMC under MDA-CM increased profoundly, while the extent of SRMC with 10A-CM was similar to that of control medium (Fig. 2d). If the inhibition of cell invasion by MDA-CM is due to an increase in cellular contractility, we anticipate that a decrease in cellular contractility induced by MDA-CM may reverse cell invasion. Figure 2e shows that the invasion of MDA-MB-231 cells in the Transwell system under the MDA-CM conditions was increased significantly with various pharmacological inhibitors for cellular contractility. Taken together, MDA-CM contains pro-contractile materials that induce the abnormal increase in cellular contractility, which might be the reason for the inhibition of cell invasion.

### Depletion of lipid components in MDA-CM induced the invasive activity of MDA-MB-231 cells

**Figure 3:**
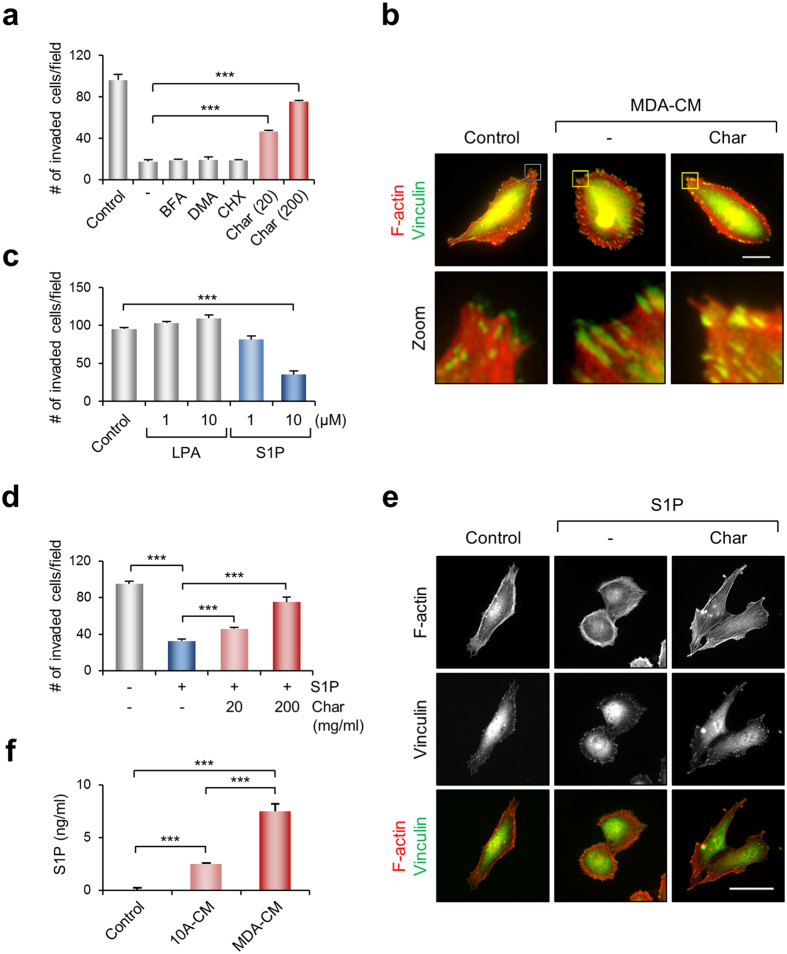
Depletion of lipid components in MDA-CM induces cell invasion. (**a**) Transwell invasion assays were performed with MDA-CM obtained from cells wherein protein secretion was blocked by treatment with various inhibitors as indicated, or depleted of lipid components by treatment with activated charcoal. Invasion values shown are the mean ± s.e.m. of triplicate samples. ***p < 0.001. (**b**) MDA-MB-231 cells incubated for 24 h with control, MDA-CM, or charcoal-treated MDA-CM, were stained for actin and the focal adhesions. Scale bar, 20 μm. (**c**) Transwell invasion assays were performed using media with serum containing LPA and S1P as indicated. Values represent mean ± s.e.m. of triplicate samples. ***p < 0.001. (**d**) Transwell invasion assays were performed using media containing S1P, which was either treated or untreated with activated charcoal as indicated. Invasion values shown are the mean ± s.e.m. of triplicate samples. ***p < 0.001. (**e**) Samples were stained for actin and focal adhesions. Scale bar, 50 μm. (**f**) Measured S1P concentration of MDA- and 10A-CM by using ELISA. Values represent mean ± s.e.m. of triplicate independent experiments. ***p < 0.001.

We examined whether the inhibition of cell invasion by MDA-CM was lost when the CM was obtained from cells in which secretion was blocked. Figure 3a showed that none of the chemical inhibitors of exocytosis (brefeldin A; BFA), exosome secretion (dimethyl amiloride; DMA), and protein synthesis (cycloheximide; CHX) rescued the inhibitory function of MDA-CM. The only exception was treatment with activated charcoal, which significantly restored the invasive ability of MDA-MB-231 cells. Lipid-depleted MDA-CM decreased the size of focal adhesions visualized by vinculin but increased cellular polarity (Fig. 3b). These results indicate that the lipid compounds secreted from MDA-MB-231 cells might be a factor that increases cellular contractility and involves an inhibitory role of the invasive ability of MDA-MB-231 cells.

Among bioactive lipids, LPA- and S1P-induced cellular contractility via RhoGTPase signaling pathway have been reported^30^,^31^. Therefore, we examined the effect of LPA or S1P on the invasion of MDA-MB-231 cells. As shown in the Fig. 3c, LPA did not inhibit the invasion of MDA-MB-231 cells induced by serum. However, medium containing S1P profoundly decreased the invasion of MDA-MB-231 cells. Depletion of S1P from the medium by activated charcoal treatment restored the serum-induced MDA-MB-231 cell invasion (Fig. 3d). Immunofluorescence images also revealed that treatment of MDA-MB-231 cells with S1P increased focal adhesions along the cell periphery, but treatment of the S1P-containing medium with activated charcoal inhibited the development of focal adhesion and cellular contractility (Fig. 3e). To compare the levels of S1P between 10A- and MDA-CM, we attempted to measure levels of S1P in the CM using ELISA^32^. Figure 3f showed that the secretion of S1P from MDA-MB-231 cells was significantly increased compared to 10A-CM (Fig. 3f). Therefore, S1P is likely to be a strong candidate among the substances secreted from MDA-MB-231 cells to induce cellular contractility and inhibit invasive activity of the cell.

### CM obtained from SphK1-silenced MDA-MB-231 cells does not inhibit invasive activity of MDA-MB-231 cells

**Figure 4:**
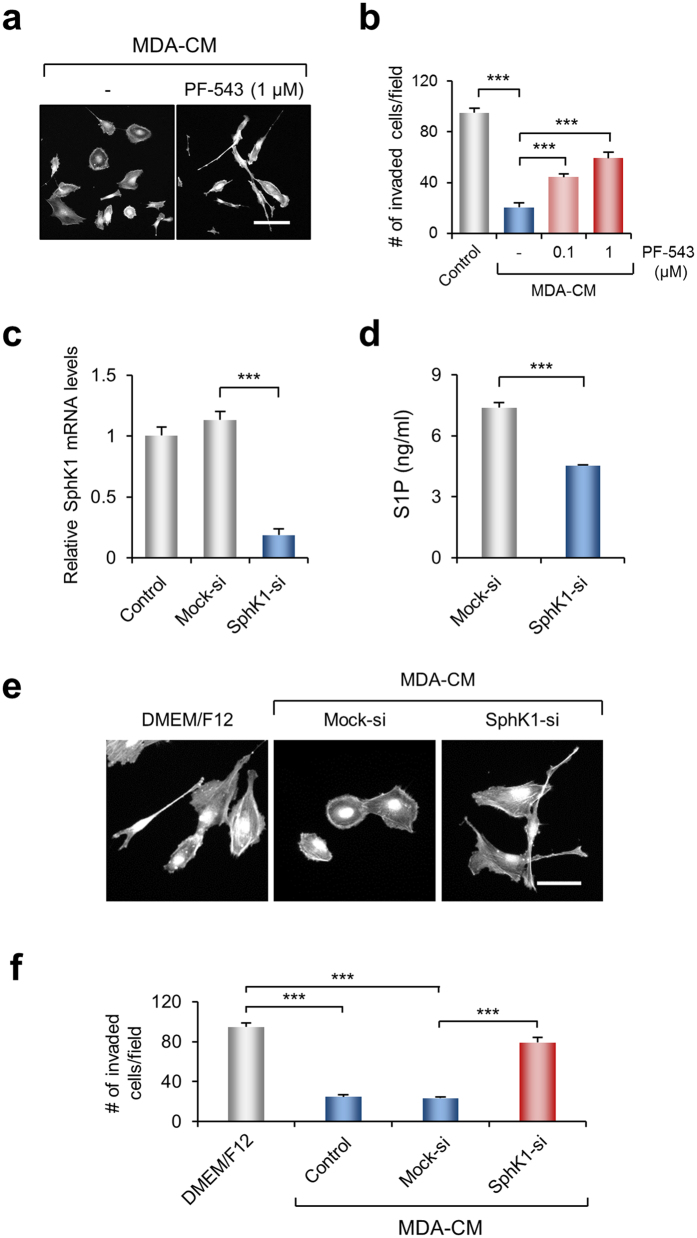
MDA-CM obtained from *SphK1* knocked-down cells does not inhibit cell invasion. (**a**) MDA-MB-231 cells were incubated for 24 h with MDA-CM obtained from PF-543-treated MDA-MB-231 cells and stained for actin. Scale bar, 100 μm. (**b**) Transwell invasion assays were performed with MDA-CM obtained from PF-543-treated cells. Values represent mean ± s.e.m. of triplicate samples. ***p < 0.001. (**c**) MDA-MB-231 cells were transfected with a 20 nM of mock or *SphK1* siRNA and incubated for 72 h. Extent of decrease of *SphK1* mRNA was quantified by real-time PCR. Values represent mean ± s.e.m. ***p < 0.001. (**d**) Measured S1P concentration of CM obtained from mock or *SphK1* siRNA-transfected cells using ELISA. Values represent mean ± s.e.m. of triplicate independent experiments. ***p < 0.001 (**e**) MDA-MB-231 cells were incubated for 24 h with MDA-CM obtained from mock or *SphK1* siRNA-transfected cells as indicated. After incubation, cells were fixed and stained for actin. Scale bar, 50 μm. (**f**) Transwell invasion assay was performed for 24 h with MDA-CM obtained from indicated condition for 24 h. Values represent mean ± s.e.m. of triplicate independent experiments. *** *p* < 0.001.

Although it has been previously reported that S1P synthesis is mediated by two sphingosine kinases (SphKs), SphK1 and SphK2, the level of extracellular S1P is regulated by SphK1 in MDA-MB-231 cells^33^. Therefore, we explored the effect on MDA-MB-231 cell invasion of the CM obtained from cells where SphK1 activity was blocked by a pharmacological inhibitor, PF-543^34^. Figure 4a shows that MDA-MB-231 cells incubated with MDA-CM obtained from PF-543-treated cells exhibited an elongated morphology with extensive lamellipodia at the leading edge; MDA-CM obtained from PF-543-treated cells did not prevent the invasive activity of MDA-MB-231 cells (Fig. 4b), which was consistent with the results obtained thus far.

To obtain more direct evidence for the requirement of SphK1 activity for the inhibitory effect of MDA-CM in cell invasion, SphK1 was silenced in MDA-MB-231 cells by siRNA transfection. Treatment of cells with siRNA decreased the *SphK1* transcript specifically without affecting the level of GAPDH transcript (Fig. 4c). The amount of S1P in the CM obtained from the *SphK1* knockdown MDA-MB-231 was reduced by 40% compared with that of Mock-siRNA-treated cells (Fig. 4d). MDA-MB-231 cells incubated with MDA-CM obtained from *SphK1*-silenced cells exhibited elongated cell bodies with massive lamellipodia compared with cells that were incubated with MDA-CM generated from mock siRNA-transfected cells (Fig. 4e). MDA-CM obtained from *SphK1*-silenced cell also did not show an inhibitory effect on MDA-MB-231 cell invasion (Fig. 4f).

### SphK1 expression is dependent on ECM rigidity

**Figure 5:**
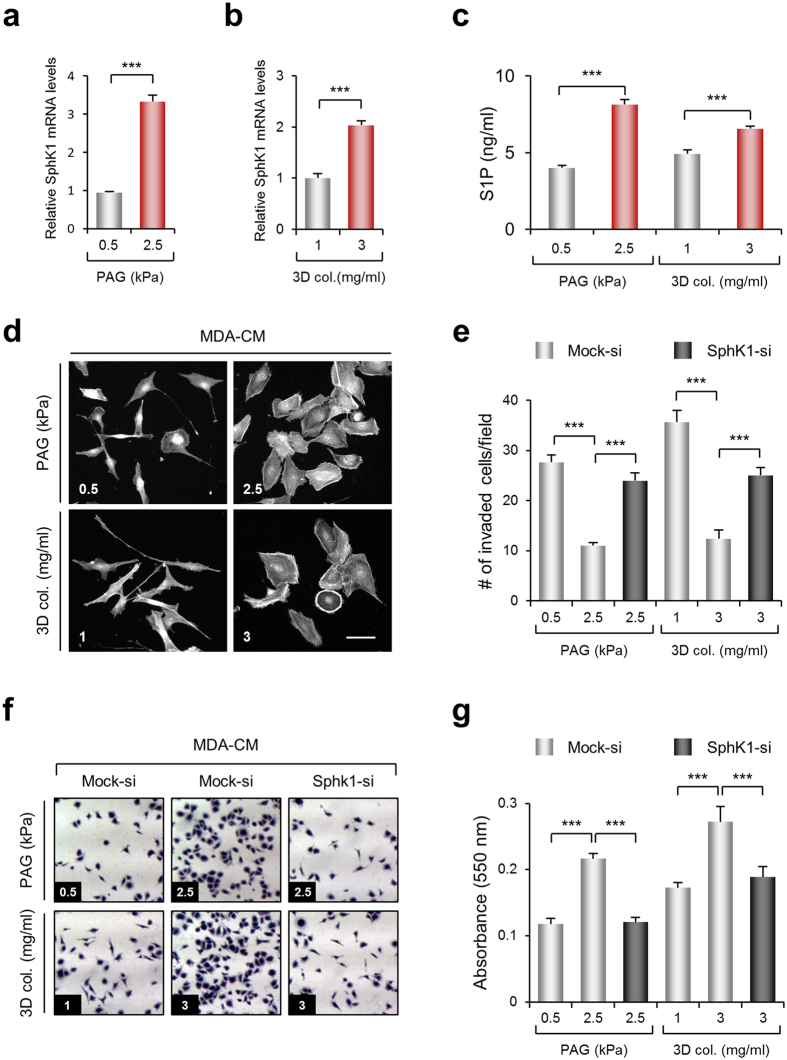
ECM rigidity-dependent *SphK1* expression affects cell invasion and adhesion. Expression of *SphK1* mRNA in MDA-MB-231 cells cultured on PAG with different rigidities as indicated (**a**) and in 3D collagen matrices with collagen concentration as indicated (**b**), were verified by real-time PCR. Values represent mean ± s.e.m. of triplicate experiments ***p < 0.001. (**c**) Measurement of S1P concentration in MDA-CM is carried out using ELISA. Values represent mean ± s.e.m. of triplicate independent experiments. ***p < 0.001 (**d**) MDA-MB-231 cells were incubated for 24 h with MDA-CM obtained from PAG or 3D collagen matrices, as indicated. After incubation, samples were stained for actin. Scale bar, 50 μm. (**e**) Transwell invasion assays were performed with MDA-CM used in (**c**). Values represent mean ± s.e.m. of triplicate independent experiments. ***p < 0.001. (**f**) MDA-MD-231 cells were plated on 96-well, 50 μg/ml collagen-coated plates and incubated for 60 min with MDA-CM obtained from various conditions as indicated. Non-adherent cells were washed out and adherent cells were fixed and stained with crystal violet. Adherent cells were photographed under a light microscope using a 10X objective lens. (**g**) Adherent cells were measured at 550 nm using a multi-plate reader. Values represent mean ± s.e.m. of triplicate independent experiments. ***p < 0.001.

Since MDA-MB-231 cells are known to possess aggressive metastatic characteristics, the fact that the secreted molecules from MDA-MB-231 rather inhibit its invasion is controversial. Interestingly, it has been previously reported that gene expression can be influenced by stiffness of the surrounding ECM environment^35^, therefore we speculated that *SphK1* expression is also controlled by matrix stiffness. To test this hypothesis, MDA-MB-231 cells were cultured on polyacrylamide gel (PAG) and 3-dimensional (3D) collagen matrices mimicking the physiological rigidities of early and late-stage breast cancer stroma, and the expression of *SphK1* mRNA was evaluated by qRT-PCR. Figure 5a shows that the relative expression of *SphK1* mRNA is much higher at 2.5 kPa PAG, which is within the stiffness range of late breast cancer tissues compared with that of normal breast tissue and early breast cancer stroma (approximately 0.5 kPa)^36^. Expression of *SphK1* mRNA in 3D collagen matrices also showed that a high-density (HD) 3D collagen matrix substantially induced *SphK1* expression compared with a low-density (LD) 3D collagen matrix (Fig. 5b). In addition, the amount of S1P secreted from MDA-MB-231 cell lines cultured on 2.5 kPa PAG and HD 3D collagen matrix was increased by 2 and 1.3 times, respectively (Fig. 5c). Morphological analysis showed that MDA-CM obtained from 0.5 kPa PAG and LD 3D collagen matrices promoted cellular polarity similar to that of cells incubated with MDA-CM obtained from *SphK1*-silenced cells, while cells incubated with MDA-CM obtained from 2.5 kPa PAG and LD 3D collagen matrices presented a more rounded morphology. This indicated that the circumstance in which the cell inhibits *SphK1* expression affects its polarity (Fig. 5d). Invasive activity of MDA-MB-231 cells also increased with MDA-CM obtained from 0.5 kPa PAG or LD 3D collagen matrices compared with that in with CM obtained from 2.5 kPa PAG or HD 3D collagen matrices. However, MDA-CM generated from the stiff environment with the *SphK1*-silenced cells did not show the inhibitory effect on invasive activity of MDA-MB-231 cells (Fig. 5e), indicating that *SphK1* expression is critical for the regulation of cell invasion, likely mediated by the S1P level.

To determine the physiological role of SphK1 and its biological product, S1P in the media, we performed the cell-matrix binding assay, since it has previously been reported that increased cellular contractility induces cell-matrix interaction^37^. MDA-CM obtained from 2.5 kPa PAG or HD 3D collagen matrix conditions promotes adhesive activity, which is reversibly decreased by *SphK1* silencing (Fig. 5f,g). Taken together, *SphK1* expression and its biological product, S1P are critical for the regulation of invasiveness and adhesiveness in response to the mechanical status in cancer tissues.

### An increase in SphK1 expression by ECM hardening is characteristic of aggressive metastatic cancer cells

We investigated the expression of SphK1 depending on the ECM stiffness using various cancer cell lines. Interestingly, normal breast epithelial cell lines, MCF10A, and primary breast cancer cell lines, Hs578T and BT20, showed the expression of *SphK1* expression in stiff substrates was decreased by 15%, 16%, and 23% compared with soft substrates, respectively (Table 1). Levels of S1P in CM were also reduced in these cells. However, cancer cells originating from pleural effusion with metastatic characteristics including MDA-MB-231 increased SphK1 expression in stiff substrates and increased S1P secretion. The exception to this was the MCF7, non-aggressive breast cancer cell lines. These results indicate that aggressive metastatic cancer cells, not primary cancer cells, increased *SphK1* expression in stiff ECM.

**Figure 6:**
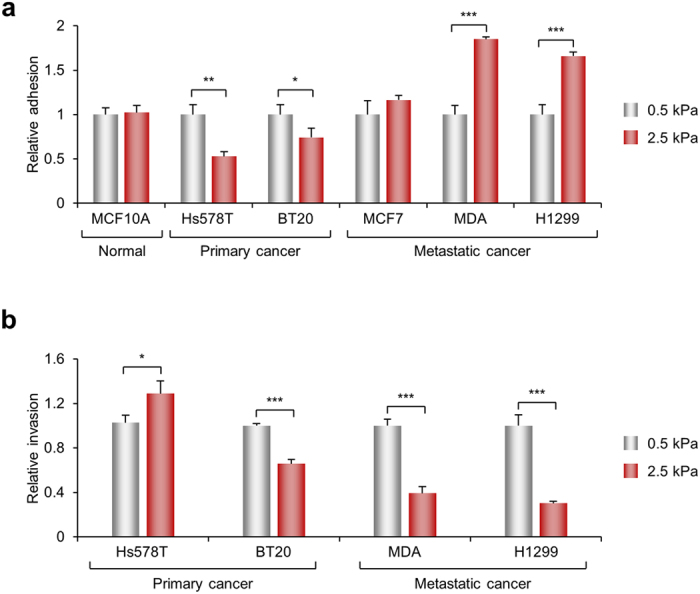
ECM rigidity-dependent CM regulates cell adhesion and invasion. (**a**) Various cells were plated on 96-well, 50 μg/ml collagen-coated plates and incubated for 60 min with CM obtained from various conditions as indicated. Non-adherent cells were washed out and adherent cells were fixed and stained with crystal violet. Adherent cells were measured at 550 nm using a multi-plate reader. Values represent mean ± s.e.m. of triplicate experiments *p < 0.05, **p < 0.01, ***p < 0.001. (**b**) Transwell invasion assay was performed with CM harvest from various cancer cells cultured at 0.5 and 2.5 kPa. Values represent mean ± s.e.m. of triplicate experiments *p < 0.05, ***p < 0.001.

Next, we investigated whether the *SphK1* expression in response to ECM stiffness influences cell invasion and adhesion (Fig. 6). MCF10A and MCF7, which did not change the SphK1 expression at different rigidities, showed no significant change in cell adhesion between CM harvested from 0.5 or 2.5 kPa PAG. CM obtained from Hs578T and BT20 cells cultured in 2.5 kPa PAG, with decreased *SphK1* expression, reduced cell adhesion while CM derived from MDA-MB-231 and H1299 cells cultured in 2.5 kPa, with increased *SphK1* expression, profoundly induced cell adhesion. Taken together, S1P secretion with an increase the *SphK1* expression in stiff substrate is characteristic of aggressive metastatic cancer cells.

## Discussion

Progression of malignant cancer is often associated with abnormal secretion of various cytokines by cancer cells, the process of which is closely related to the development of cancer, and influenced by the surrounding cells in an autocrine and a paracrine manner. For example, increased secretion of TGF-β can lead to epithelial-mesenchymal transition (EMT), a developmental process that forces metastatic properties upon cancer cells, facilitating migration and invasion^38^. Moreover, cancer cells trigger angiogenesis and enhance growth via secretion of vascular endothelial growth factor (EGF) and platelet-derived growth factor (PDGF). However, our data showed that MDA-CM inhibited its own cell migration and invasion. Cells treated with MDA-CM showed reduced polarity compared with plain media or CM obtained from a normal breast epithelial cell line, MCF10A. This is consistent with findings from previous studies showing growth factors secreted from normal breast epithelial cells can induce cell migration^39^. Moreover, CM obtained from a non-small lung metastatic cancer cell line, H1299, also exhibited similar results, where cell migration and invasion induced by serum is profoundly inhibited (Supplementary Fig. S2). Thus, these results indicate that most secreted molecules from cancer cells may not be involved in cell migration and invasion. Rather, the molecules secreted from cancer cells are responsible for cell survival and growth^40^,^41^.

Our observation that MDA-CM induced focal adhesion formation and the phosphorylation of FAK and myosin light chain indicates that MDA-CM contains a potent agonist for cell contraction. Furthermore, an increase in SRMC supports the idea that molecules secreted from MDA-MB-231 cells have the potential to enable cell contraction instead of cell migration, since SRMC is often used as a model for cell contraction^42^. Although it was previously reported that cellular contractility accompanying tyrosine phosphorylation of FAK and Src kinase is critical for cell migration^43^, the spatio-temporally optimal level for cellular contractility is indispensable for cell migration^44^. Thus, our results suggest that maximal activation of cellular contractility by MDA-CM rather exerts an inhibitory effect on cell migration and invasion. Consistent with this possibility, we found that various pharmacological inhibitors for cellular contractility restored the inhibition of MDA-MB-231cell invasion by MDA-CM.

Previous reports indicate that the role of S1P in cell motility in MDA-MB-231 cells is independent of cell surface receptors, as there is no corresponding receptor on MDA-MB-231 cells^27^. Moreover, the effective range of S1P on chemotactic migration of MDA-MB-231 cells is >10 μM, which is far beyond that of S1P receptor affinity. Wang *et al*. proposed the involvement of a non-oscillatory increase in free calcium by S1P as a plausible mechanism for the its inhibitory role in cell motility, independent of receptor activity^27^. Our results also showed that treatment with ML-7, an inhibitor of myosin light-chain kinase (MLCK), did not inhibit MDA-CM-mediated cell invasion, indicating that calcium-mediated signaling such as MLCK activation is required for S1P-mediated cell motility^45^.

Next, we showed that expression levels of *SphK1* mRNA, which is a primary regulator of S1P synthesis in the cell is dependent on the mechanical status of the cell’s surrounding environment. A positive correlation is known to exist between *SphK1* expression and cancer progression. Increased *SphK1* expression has been observed in breast cancer^46^. However, in MDA-MB-231 human breast cancer cell lines, *SphK1* expression was not observed in this study in cells with approximately 0.5 kPa stiffness, which is similar to the rigidity of normal breast tissue, and was profoundly increased in cells with approximately 2.5 kPa stiffness, corresponding to breast cancer stiffness^11^. Moreover, MDA-CM obtained with 0.5 kPa stiffness did not interfere with cell invasion induced by serum, as opposed to CM obtained from cells with 2.5 kPa stiffness. It has been previously reported that the genetic and epigenetic alterations, along with changes in the biophysical properties of fibrotic stroma surrounding the tumor cell gradually occur as tumorigenesis proceeds^47^. For example, expression of Siva1, which acts as negative regulator of EMT, and post-translational modification of stathmin, a microtubule destabilizer, are different in primary tumors and metastatic tumors in breast cancer patients^48^. In addition, in pancreatic and breast cancer cells, MMP activity, which depends on the stiffness of ECM, differs between metastatic and primary cancer cells^49^. In this regard, the expression of *SphK1* is likely one of markers for the mechano-responsive genes for tumorigenesis.

In addition, regulation of expression of *SphK1* and S1P secretion appeared to be influenced by factors other than matrix stiffness. Since expression of SphK1 in the triple negative (TN) cancer cell line, MDA-MB-231 was upregulated in stiff substrate in our results, we anticipated that breast cancer cells would increase SphK1 levels in stiff substrates. However, several breast cancer cell lines including TN breast cancer cell lines such as Hs578T and BT20 decreased the *SphK1* expression in stiff substrate. Thus, there might be another factor involved in the regulation of SphK1 expression in addition to matrix stiffness. When results were analyzed regarding cells as primary or metastatic cancer cells based on their origin, we found that cancer cells taken from primary tumor sites tend to show a decrease in *SphK1* expression in stiff substrate, whereas cancer cells taken from metastatic sites (e.g. MDA-MB-231 from pleural effusion; H1299 from lymph nodes), showed an increase in *SphK1* expression in stiff substrate. Thus, these results indicate that *SphK1* expression in response to ECM stiffness is likely to be determined by the metastatic status of cells.

Considering the seed and soil hypothesis, cancer progression is closely related with the mechanical properties of surrounding microenvironment^50^. During the early stages of tumorigenesis, stroma create an environment permissive of cell proliferation and invasion with minor changes to the biophysical properties of ECM. Therefore, one of the important roles for cancer cells existing in the primary site of the tumor is to change the mechanical properties of the ECM surrounding the cancer cells to generate the permissive microenvironment^51^,^52^. Our results showing that cancer cells obtained from primary tumor sites increase *SphK1* expression in soft matrix support the concept that S1P secreted by cancer cells originating from the primary site into the surrounding cells and tissue allows cells to develop a mechanically permissive environment for cancer proliferation and survival. However, metastatic cancer cells must colonize once they extravasate from blood vessels, and the cells also require the ability to promote adhesion between the cells and the substrate^53^. We also showed that MDA-MB-231 and H1299, the aggressive metastatic cancers, increased *SphK1* expression and adhesive activity in the stiff substrate. Normally, breast and lung cancer cell metastasizes to the bone and liver^54^,^55^, in which mechanical properties are stiffer than those of the primary site. Metastatic cancer cell originating from breast and lung must increase the adhesive activity once they arrive at the stiff substrate^56^. Thus, it is plausible that the increase in expression of *SphK1* on stiff substrate may be a critical property of a metastatic cancer cell for facilitating the cell-matrix adhesion. In this regard, development of a pharmacological inhibition therapy targeting SphK1 is a useful therapeutic strategy for both early and late stages of cancer, although no specific inhibitors targeting SphK1 have been developed as yet^57^.

## Methods

### Cell culture

MDA-MB-231, Hs578T, BT20, MCF7, and H1299 cells were purchased from Korea Cell Line Bank (Seoul, Korea). MCF10A cells were obtained from the American Type Culture Collection (Manassas, VA, USA). MDA-MB-231, BT20 and MCF7 cells were maintained in RPMI 1640 medium (Invitrogen, Carlsbad, CA, USA), supplemented with 10% (v/v) fetal bovine serum (FBS; Biowest, Nuaillè, France), 100 units/mL penicillin, and 100 μg/mL streptomycin (Welgene, Korea). Hs578T cells were maintained in Dulbecco’s Modified Eagle’s Medium (Invitrogen, Carlsbad, CA, USA), supplemented with 10% (v/v) FBS, 100 units/mL penicillin, and 100 μg/mL streptomycin. MCF10A cells were cultured in DMEM/F12 medium (Invitrogen, Carlsbad, CA, USA) containing 10 μg/mL bovine insulin, 20 ng/mL epidermal growth factor, 100 ng/mL cholera enterotoxin, 0.5 μg/mL hydrocortisone (Sigma, St. Louis, MO, USA), and 5% horse serum (Welgene, Korea).

### Preparation of conditioned media

MDA-MB-231, MCF10A, Hs578T, BT20, MCF7, and H1299 cells were grown in culture media until the cell density reached 70 to 80% confluence. The cells were then washed with DMEM/F12 thrice, before they were incubated in DMEM/F12 at 37 °C for 24 h. After incubation, CM was harvested, centrifuged, and filtered through a 0.2 μm filter.

### Cell invasion through Transwells

Transwells (Costar, Corning, NY, USA) with 8 μm pore size were coated with 10 μg type I collagen (BD Bioscience, Bedford, MA, USA) overnight, at 37 °C. Cells were seeded in the upper chamber and DMEM/F12 or CM supplemented with 10% FBS was added to the lower chamber. Non-invading cells were removed with a cotton swab. Cells were fixed and stained with 0.1% crystal violet. The number of invading cells was quantified by counting of least five random fields per filter.

### Preparation of polyacrylamide gels

Polyacrylamide gels (PAG) with various degrees of stiffness were prepared as previously described^58^. Acrylamide and bis-acrylamide were mixed to obtain desired concentrations in distilled water and a thin layer was applied on coverslips treated with 2% 3-aminopropyl-trimethoxysilan. The polyacrylamide gel was treated with 0.5 mg/mL sulfo-sulfosuccinimidyl-6-[4′-azido-2′-nitrophenylamino] hexanoate (Sigma, St. Louis, MO, USA) and exposed twice, to 365 nm UV light for 14 min each. Coverslips were coated with 50 μg/ml type I collagen for 16 h at 4 °C. Before cell seeding, coverslips were washed with phosphate-buffered saline (PBS) and exposed to UV light for 15 min. The stiffness of PAG was characterized by Atomic Force Microscopy (NX10, Park systems Corp., Suwon, Korea).

### siRNA transfection

MDA-MB-231 cells were incubated with transfection media containing 20 nM siRNA and oligofectamine (Invitrogen) transfection reagent for 24 h and replaced with RPMI 1640 supplemented with 10% FBS for an additional 48 h. *SphK1* siRNA (5′- GGGCAAGGCCUUGCAGCUC-3′), and a control mock siRNA (5′-AUUGUAUGCGAUCGCAGAC-3′) were used.

### Quantitative reverse-transcription polymerase chain reaction (qRT-PCR) analysis

Total RNA was obtained using RNAiso Plus (TaKaRa Bio, Tokyo, Japan) according to the manufacturer’s protocol. cDNA synthesis was carried out using M-MLV (M. biotech, Seoul, Korea) reverse transcriptase according to the manufacturer’s instructions. The PCR reaction conditions were as follows: *SphK1*, GAPDH initial denaturation at 95 °C for 3 min, followed by 40 cycles at 95 °C for 10 sec, 57 °C for 10 sec, and 72 °C for 30 sec. The human *SphK1* primer sequences were 5′-ATGCACGAGGTGGTGAACG-3′ (sense) and 5′-GGAGGCAGGTGTCTTGGAAC-3′ (antisense), and the human GAPDH primer sequences were 5′-ACCCAGAAGACTGTGGATGG-3′ (sense) and 5′-TTCTAGACGGCAGGTCAGGT-3′ (antisense).

### Western blot analysis

Cells were homogenized in lysis buffer comprising 150 mM NaCl, 6 mM Na_2_HPO_4_, 4 mM NaH_2_PO_4_, 2 mM EDTA, 1% sodium deoxycholate, 1% nonidet P-40, 50 mM NaF, 2% sodium dodecyl sulfate (SDS), 1 mM Na_3_VO_4_, and 10 mM phenylmethylsulfonyl fluoride. Prepared samples were subjected to SDS-polyacrylamide gel electrophoresis (PAGE) and then transferred to a PVDF membrane (Millipore, Bedford, MA, USA). The membranes were blocked, washed, and incubated with primary and secondary antibodies. The signal was developed with an enhanced chemiluminescence reagent.

### Immunofluorescence staining

Cells were seeded on 50 μg/mL type I collagen-coated, 12-mm coverslips and fixed for 15 min with 3.7% paraformaldehyde, permeabilized for 10 min with 0.5% Triton X-100 in PBS, and blocked with 2% bovine serum albumin (BSA) in 0.1% Triton X-100-containing PBS for 1 h. Samples were then incubated for 1 h with mouse anti-vinculin (1:100 dilution in 2% BSA/PBS), rabbit anti-FAK (1:100 dilution in 2% BSA/PBS), or rabbit anti-phospho FAK (1:100 dilution in 2% BSA/PBS) antibodies, followed by incubation for 1 h with fluorescein isothiocyanate-conjugated, goat anti-mouse IgG or goat anti-rabbit IgG. For actin staining, samples were incubated with Alexa Fluor 594–conjugated phalloidin (1:100 dilution in PBS containing 0.1% Triton X-100) for 1 h. Samples were mounted on glass slides with Fluoromount-G (Southern Biotechnology Associates, Birmingham, AL). Images were obtained using a fluorescence microscope (Eclipse 80i, Nikon, JAPAN) with Plan Fluor 10 × /0.30, Plan Apo 20 × /0.75, and Plan Fluor 40 × /0.75 infinity-corrected objectives. Images were captured using a digital camera (digital sightDS-Qi1Mc, Nikon) and NIS elements image analysis software (Nikon). Image processing was carried out using Photoshop 11.0 (Adobe Systems, San Jose, CA, USA).

### Stress-released matrix contraction

Collagen matrices (1.5 mg/mL collagen, 2 × 10^5^ cells/matrix) were polymerized for 1 h at 37 °C and matrices were cultured for 24 h in DMEM/F12 supplemented with 10% FBS. After incubation and rinsing with PBS, the matrices were released from the plate and incubated for 1 h while floating in CM, as shown in figure 2e. At the end of incubation, matrices were fixed with 3.7% paraformaldehyde and matrix diameters were measured.

### S1P ELISA

S1P concentrations in CM were measured using an S1P ELISA kit (MyBioSource, San Diego, USA). All procedures were performed according to the manufacturer’s protocols. S1P concentrations were quantified in triplicate and compared with an internal S1P standard (3.125~25 ng/mL). Optical Density (O.D.) at 450 nm was measured by using microplate reader.

### Statistical analysis

Differences between control and treatment groups were analyzed by the Student’*s t-test* using GraphPad PRISM software (Graphpad Software, San Diego, CA, USA). The data are expressed as average ± s.e.m. of three independent experiments. A value of *p* < 0.05 was considered to be the threshold of significance.

## Additional Information

**How to cite this article**: Ko, P. *et al*. Extracellular Matrix Rigidity-dependent Sphingosine-1-phosphate Secretion Regulates Metastatic Cancer Cell Invasion and Adhesion. *Sci. Rep.* **6**, 21564; doi: 10.1038/srep21564 (2016).

## Supplementary Material

Supplementary Information

## Figures and Tables

**Table 1:** Relative expression of SphK1 and extracellular S1P (2.5 kPa/0.5 kPa CM) in various cell lines.

	Cell lines	SphK1	S1P
Normal cell	MCF10A	0.9	1.1
Primary cancer cell	Hs578T	0.8*	0.6*
	BT20	0.8*	0.4**
Metastatic cancer cell	MCF7	1.0	1.0
	MDA-MB-231	3.3***	2.0***
	H1299	1.4**	2.6***

Data obtained at 2.5 kPa differs compared to data obtained at 0.5 kPa. The Student’s *t-test* was used for statistical analysis. *p < 0.05, **p < 0.01, ***p < 0.001.
