# Supplementary material for: Extracellular Matrix Rigidity-dependent Sphingosine-1-phosphate Secretion Regulates Metastatic Cancer Cell Invasion and Adhesion
Source: Sci Rep. 2016 Feb 15;6:21564. doi: 10.1038/srep21564 (PMC4753492; doi:10.1038/srep21564)
Supplement: Supplementary Information [file srep21564-s1.pdf]

Supplementary information

Supplementary Figure S1 – S2 – S3

Extracellular Matrix Rigidity-dependent Sphingosine-1-phosphate Secretion Regulates Metastatic Cancer Cell Invasion and Adhesion

Panseon Ko, Daehwan Kim, Eunae You, Jangho Jung, Somi Oh, Jaehyun Kim, Kwang-Ho Lee, and Sangmyung Rhee*

Department of Life Science, Chung-Ang University, Seoul, 06974, Republic of Korea

*To whom correspondence should be addressed:

Tel: +82-2-820-5818; Fax: +82-2-825-5206

E-mail: Sangmyung.rhee@cau.ac.kr

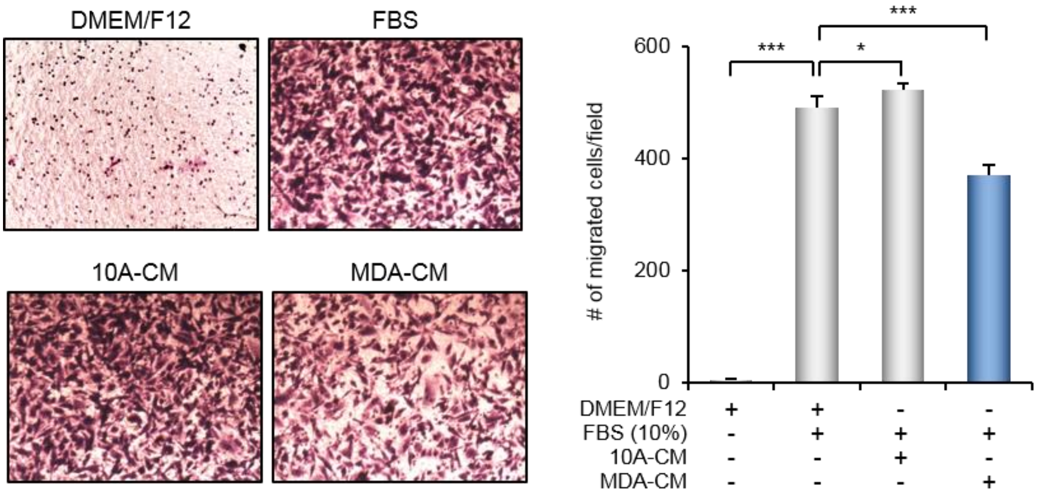

Supplementary Figure S1. MDA-CM inhibits serum-induced chemo-attractive migration. Migration of MDA-MB-231 cells was examined with MDA- or 10A-CM using a non-collagen-coated Transwell migration assay system for 24 h. Right panels, quantification of the average number of migrated cells per photographic field shown in the left panel. Values represent mean ± SEM, * $p<0.05$, *** $p<0.001$.

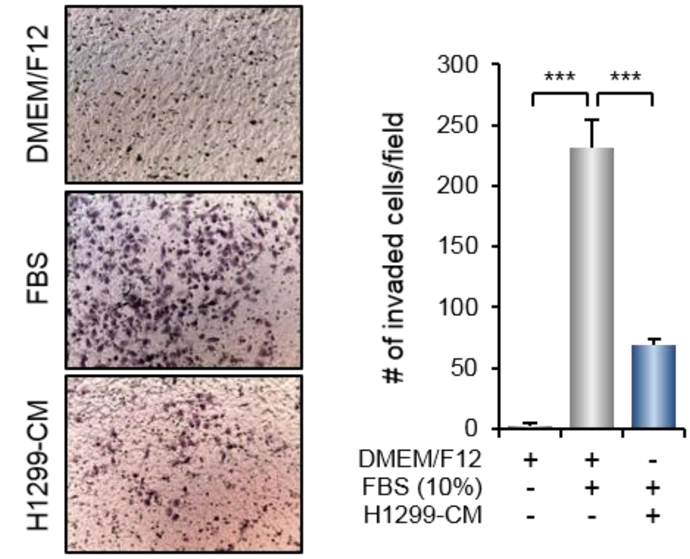

Supplementary Figure S2. H1299-CM inhibits serum-induced chemo-attractive invasion. Invasion of H1299 cells was examined with H1299-CM using a collagen-coated Transwell invasion assay system for 24 h. Right panels, quantification of the average number of invading cells per photographic field shown in the left panel. Values represent mean ± SEM, *** *p*<0.001.

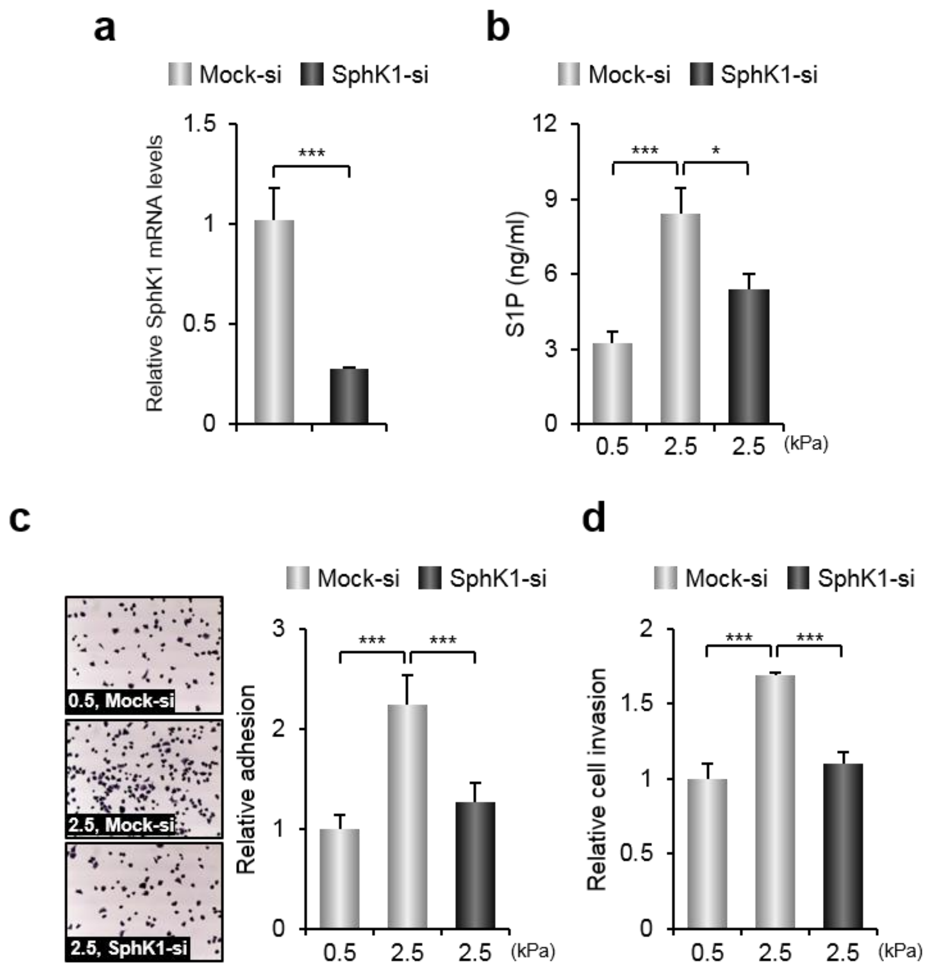

Supplementary Figure S3. ECM rigidity-dependent SphK1 expression affects cell adhesion and invasion in H1299. (*a*) H1299 cells were transfected with a 20 nM of mock or *SphK1* siRNA and incubated for 72 h. Extent of decrease of *SphK1* mRNA was quantified by real-time PCR. Values represent mean ± SEM. *** $p<0.001$. (*b*) Measurment of extracellular S1P from H1299 at 0.5 and 2.5 kPa PAG. (*c*) Adhesions of H1299 were calculated relative to 0.5 kPa CM. (*d*) Transwell invasion assays were performed with CM harvest from H1299 cultured at 0.5 and 2.5 kPa. Values represent mean ± SEM of triplicate experiments * $p<0.05$, ***$p<0.01$.
